# Supplementary material for: PHEIGES: all-cell-free phage synthesis and selection from engineered genomes
Source: Nat Commun. 2024 Mar 12;15:2223. doi: 10.1038/s41467-024-46585-1 (PMC10933291; doi:10.1038/s41467-024-46585-1)
Supplement: Supplementary file 3 — Description of Additional Supplementary Files [file 41467_2024_46585_MOESM3_ESM.pdf]

Description of Additional Supplementary Files

Supplementary Movie 1 - Timelapse image sequence of E. coli B cells infected with T7-mC-gp10.

Supplementary Datasets

Supplementary Data 1: Fragment sequences of T7 phage genomes engineered in this work and rebooted Felix01,

Supplementary Data 2: Primers used to assemble T7 genomes in this work as well as the assembly maps,

Supplementary Data 3: Mutations table from all the fully sequenced phages in this work,

Supplementary Data 4: Plasmids used in this work,

Supplementary Data 5: Comparison of the tail and tail fiber mutations leading to ReLPS strains infection with other work.

Supplementary Data 6: Correspondence between accession number and sample.
