# Supplementary material for: PHEIGES: all-cell-free phage synthesis and selection from engineered genomes
Source: Nat Commun. 2024 Mar 12;15:2223. doi: 10.1038/s41467-024-46585-1 (PMC10933291; doi:10.1038/s41467-024-46585-1)
Supplement: Supplementary file 6 — Reporting Summary [file 41467_2024_46585_MOESM6_ESM.pdf]

nature portfolio

Corresponding author(s): Ariel Lindner, Vincent Noireaux

Last updated by author(s): Feb 26, 2024

# Reporting Summary

Nature Portfolio wishes to improve the reproducibility of the work that we publish. This form provides structure for consistency and transparency in reporting. For further information on Nature Portfolio policies, see our Editorial Policies and the Editorial Policy Checklist.

## Statistics

For all statistical analyses, confirm that the following items are present in the figure legend, table legend, main text, or Methods section.

| n/a | Confirmed | |
|---|---|---|
| ☐ | ☒ | The exact sample size (*n*) for each experimental group/condition, given as a discrete number and unit of measurement |
| ☐ | ☒ | A statement on whether measurements were taken from distinct samples or whether the same sample was measured repeatedly |
| ☒ | ☐ | The statistical test(s) used AND whether they are one- or two-sided<br>*Only common tests should be described solely by name; describe more complex techniques in the Methods section.* |
| ☒ | ☐ | A description of all covariates tested |
| ☒ | ☐ | A description of any assumptions or corrections, such as tests of normality and adjustment for multiple comparisons |
| ☐ | ☒ | A full description of the statistical parameters including central tendency (e.g. means) or other basic estimates (e.g. regression coefficient) AND variation (e.g. standard deviation) or associated estimates of uncertainty (e.g. confidence intervals) |
| ☒ | ☐ | For null hypothesis testing, the test statistic (e.g. *F*, *t*, *r*) with confidence intervals, effect sizes, degrees of freedom and *P* value noted<br>*Give P values as exact values whenever suitable.* |
| ☒ | ☐ | For Bayesian analysis, information on the choice of priors and Markov chain Monte Carlo settings |
| ☒ | ☐ | For hierarchical and complex designs, identification of the appropriate level for tests and full reporting of outcomes |
| ☒ | ☐ | Estimates of effect sizes (e.g. Cohen's *d*, Pearson's *r*), indicating how they were calculated |

*Our web collection on statistics for biologists contains articles on many of the points above.*

## Software and code

Policy information about availability of computer code

| | |
|---|---|
| Data collection | Agilent Biotek Gen 5 software (plate reader data), Metamorph (microscopy), Fiji (Microscopy) |
| Data analysis | Kaleidagraph (Synergy), elementary Python codes for sequence analysis and Galaxy software for NGS analysis. |

For manuscripts utilizing custom algorithms or software that are central to the research but not yet described in published literature, software must be made available to editors and reviewers. We strongly encourage code deposition in a community repository (e.g. GitHub). See the Nature Portfolio guidelines for submitting code & software for further information.

## Data

Policy information about availability of data

All manuscripts must include a data availability statement. This statement should provide the following information, where applicable:

- Accession codes, unique identifiers, or web links for publicly available datasets
- A description of any restrictions on data availability
- For clinical datasets or third party data, please ensure that the statement adheres to our policy

Source Data are provided with this paper. The data set used in the main Figures are presented as a Source data excel document. All Source Data necessary to reproduce analysis and plots of this paper are also available on the GitHub repository: https://github.com/Noireauxlab-TXTL/PHEIGES. Supplementary Source Data containing Source Microscopy Data are provided on the University of Minnesota Digital Conservancy (https://conservancy.umn.edu/) with the permanent URL: https://hdl.handle.net/11299/260237, and under public available license. The engineered T7 genome sequences generated in this study are available in the

GenBank database under consecutive accession codes PP384393 to PP384410 (correspondence table provided in Supplementary Data 6). The mutated tip of tail fiber sequences generated in this study are available in the GenBank database under consecutive accession codes PP379475 to PP379532 (correspondence table provided in Supplementary Data 6). The raw genome data generated in this study are available in the SRA database under accession code PRJNA1077253 (https://www.ncbi.nlm.nih.gov/sra/PRJNA1077253). The raw NGS tail fiber data used in this study are available in the SRA database under accession code PRJNA1077490 (https://www.ncbi.nlm.nih.gov/sra/PRJNA1077490).

## Research involving human participants, their data, or biological material

Policy information about studies with human participants or human data. See also policy information about sex, gender (identity/presentation), and sexual orientation and race, ethnicity and racism.

| | |
|---|---|
| Reporting on sex and gender | Our work does not contain any human participants. |
| Reporting on race, ethnicity, or other socially relevant groupings | Our work does not contain any human participants. |
| Population characteristics | Our work does not contain any human participants. |
| Recruitment | Our work does not contain any human participants. |
| Ethics oversight | Our work does not contain any human participants. |

Note that full information on the approval of the study protocol must also be provided in the manuscript.

# Field-specific reporting

Please select the one below that is the best fit for your research. If you are not sure, read the appropriate sections before making your selection.

☒ Life sciences ☐ Behavioural & social sciences ☐ Ecological, evolutionary & environmental sciences

For a reference copy of the document with all sections, see nature.com/documents/nr-reporting-summary-flat.pdf

# Life sciences study design

All studies must disclose on these points even when the disclosure is negative.

| | |
|---|---|
| Sample size | Around 50 tail fiber gene sequences were sequenced to assess the mutation landscape. For NGS analysis, we used a large sample size (600 000 reads) to evaluate the mutation rate. For mutation landscape, a small subset (50 tail fibers) were sequenced to verify the mutations. |
| Data exclusions | No data points were excluded. |
| Replication | At least triplicates for cell-free gene expression reactions. All attempts at replication were successful. |
| Randomization | Our research aimed at exploring new engineering ideas and proving concepts. Randomization was not prioritized as this small-scale study focused on generating preliminary data statistically valid. |
| Blinding | We prioritized concept validations. Blinding would be used at later stages for broader validation, especially considering the added complexity and resource implications. |

# Reporting for specific materials, systems and methods

We require information from authors about some types of materials, experimental systems and methods used in many studies. Here, indicate whether each material, system or method listed is relevant to your study. If you are not sure if a list item applies to your research, read the appropriate section before selecting a response.

### Materials & experimental systems

| n/a | Involved in the study | |
|---|---|---|
| ☒ | ☐ | Antibodies |
| ☒ | ☐ | Eukaryotic cell lines |
| ☒ | ☐ | Palaeontology and archaeology |
| ☒ | ☐ | Animals and other organisms |
| ☒ | ☐ | Clinical data |
| ☒ | ☐ | Dual use research of concern |
| ☒ | ☐ | Plants |

### Methods

| n/a | Involved in the study | |
|---|---|---|
| ☒ | ☐ | ChIP-seq |
| ☒ | ☐ | Flow cytometry |
| ☒ | ☐ | MRI-based neuroimaging |

## Plants

| | |
|---|---|
| Seed stocks | NA |
| Novel plant genotypes | NA |
| Authentication | NA |
